# Protective Effect of High-Intensity Interval Training (HIIT) and Moderate-Intensity Continuous Training (MICT) against Vascular Dysfunction in Hyperglycemic Rats

**DOI:** 10.1155/2022/5631488

**Published:** 2022-12-03

**Authors:** Nurul Paramita, Brilliant C. Puspasari, Randika Arrody, Neng T. Kartinah, Trinovita Andraini, Julfiana Mardatillah, Hardiyanti Rusli, Dewi I. S. Santoso

**Affiliations:** ^1^Department of Medical Physiology and Biophysics, Faculty of Medicine, Universitas Indonesia, Jakarta, Indonesia; ^2^Graduate Students of Magister Program in Biomedical Sciences, Faculty of Medicine Universitas Indonesia, Jakarta, Indonesia

## Abstract

**Background:**

Hyperglycemia is a major risk factor for endothelial dysfunction. Endothelial dysfunction is associated with the inability of endothelial cells to maintain homeostasis of the cardiovascular system. Regular exercise may be considered as an effective and low-cost nonpharmacological tool for improving vascular function, though there is no agreement on the best type of exercise.

**Objectives:**

To determine how high-intensity interval training (HIIT) and moderate-intensity continuous training (MICT) may prevent endothelial dysfunction under hyperglycemic conditions, and to compare these two interventions.

**Method:**

Twenty-four eight-week-old male Wistar rats were randomly assigned into four groups: healthy nonexercising control (C), hyperglycemic control (HG-C), hyperglycemic + HIIT (HG-IT), and hyperglycemic + MICT (HG-CT). Hyperglycemia was induced by a single injection of streptozotocin. Hyperglycemic animals were subjected to HIIT or MICT protocols six days a week for six weeks. Decapitation was performed the day after the exercise protocols were completed. The ascending aorta (until the abdominal artery) was examined. An enzyme-linked immunosorbent assay (ELISA) was used to measure the glucagon-likepeptide-1 (GLP-1), endothelial nitric oxide synthase (eNOS), and tumor necrosis factor-alpha (TNF*α*) levels. A colorimetric assay was used to measure superoxide dismutase (SOD) activity and malondialdehyde (MDA) levels. Quantitative real-time polymerase chain reaction (PCR) was used to measure the expression of the receptor for advanced glycation end-products (RAGE) and nuclear factor kappa-light-chain-enhancer of activated B cells (NF-*κ*B). Hematoxylin and eosin (H&E) staining was used to histologically analyze the aortas.

**Results:**

There was a significantly higher level of GLP-1 and lower expression of RAGE, NF-*κ*B, and TNF*α* in the HG-IT and HG-CT group compared to the HG-C group. Microscopic examination of aortic tissue showed a better tissue arrangement in both treatment groups than in the HG-C group. Except for the MDA level, there were no significant differences in any of the measured parameters between the HG-IT and HG-CT groups.

**Conclusion:**

Under hyperglycemic conditions, both HIIT and MICT have a protective role against endothelial dysfunction.

## 1. Introduction

Diabetes mellitus (DM) is characterized by high blood sugar levels that are accompanied by impaired carbohydrate, lipid, and protein metabolism as a result of insulin function insufficiency [1]. Data from the International Diabetes Federation (IDF) Diabetic Atlas showed that in 2019, 463 million people worldwide had DM, and the number is expected to increase to 700 million by 2045 [2].

DM poses a significant problem in the health sector and is associated with an increased risk of cardiovascular disease. Type 1 and type 2 DM are both significant risk factors for stroke, atherosclerosis, coronary artery disease (CAD), and peripheral arterial disease (PAD), which are responsible for over 80% of all deaths of people with DM [3]. Cardiovascular complications are the main cause of mortality in patients with type 2 DM (T2DM), and the development of cardiovascular complications caused by DM is triggered by endothelial dysfunction [4].

Vascular endothelial cells make a considerable contribution to the maintenance of cardiovascular system homeostasis through the secretion of various vasoactive agents. These include prostaglandin I_2_, endothelium-derived hyperpolarizing factor, nitric oxide (NO), angiotensin II, thromboxane A2, and endothelin-1 (ET-1). Disturbances in endothelial cells could lead to endothelial dysfunction, a condition marked by reduced NO activation or decreased NO production by endothelial nitric oxide synthase (eNOS) in endothelial cells [4].

Chronic hyperglycemia is now recognized as a key contributor to the development of vascular dysfunction in patients with DM. Hyperglycemia causes several changes in vascular tissue at the cellular level, all of which could accelerate the development of diabetic complications. The three known main pathways in the pathogenesis of organ disorders under hyperglycemic conditions are the polyol pathway, the formation of advanced glycation end-products (AGEs) followed by overexpression of the receptor for AGEs (RAGE) gene, and activation of the protein kinase C (PKC) isoform via diacylglycerol (DAG) synthesis [5]. These pathways contribute to creating oxidative stress through increased production of reactive oxygen species (ROS), such as lipid peroxidase products, and decreased production of antioxidants, such as superoxide dismutase (SOD). This results in inflammation that leads to organ dysfunction [5, 6].

The interaction between AGEs and RAGE can activate many signaling pathways, one of which is the release of proinflammatory mediators that trigger the accumulation of intracellular ROS. The accumulation of ROS also activates the proinflammatory agent tumor necrosis factor-alpha (TNF*α*), which further induces nuclear factor kappa-light-chain-enhancer of activated B cells (NF-*κ*B) [7, 8]. NF-*κ*B activation is essential for early events of atherosclerotic lesion formation [9]. NF-*κ*B acts as a transcription factor that regulates the expression of hundreds of genes that regulate critical physiological processes such as inflammation, immunity, proliferation, and cell death. NF-*κ*B controls both innate and adaptive immune response that plays a significant role in the initiation and development of atherosclerosis [10, 11]. T cells, and macrophages, for instance, initiate activation of interferon (IFN)-*γ*, a key trigger for the inflammation process that leads to atherosclerosis formation [12].

There is great interest within the health industry in developing ways to manage endothelial dysfunction under hyperglycemic conditions to prevent cardiovascular complications. The potential of incretin hormones (intestine secretion insulin), such as glucagon-likepeptide-1 (GLP-1), is one of the recent findings. GLP-1 can increase the synthesis and phosphorylation activity of eNOS and thus increase NO production. GLP-1 also inhibits NF-*κ*B activation through suppression of RAGE expression by increasing intracellular cAMP. This entails reducing binding between AGEs and RAGE, thereby reducing inflammation and promoting endothelial regeneration. The endothelial repair process prevents changes in blood vessel structure, such as the thickening of the tunica intima-media [13–16]. However, where and how GLP-1 affects the structure and function of vessels in hyperglycemic conditions have yet to be investigated.

Several studies have shown that exercise is a crucial intervention to prevent cardiovascular system complications associated with DM. In diabetic patients with insulin deficiency, regular physical exercise has been shown to facilitate the entry of glucose into muscle cells, thereby increasing insulin sensitivity. Sports activities can increase glucose-carrying protein levels, thereby reducing insulin resistance [17].

According to a study by Gibala et al. [18], exercise intensity is the key factor influencing peroxisome proliferator-activated receptor gamma coactivator 1-alpha (PGC-1*α*) activation in human skeletal muscle. The positive effects of the PGC-1*α* activation pathway include increased oxygen uptake, formation of antioxidants, increased oxidative capacity, and activation of anti-inflammatory pathways. Physical exercise also increases angiogenesis and accelerates blood flow, leading to reduced vasoconstriction and the occurrence of hypoxia [19]. Physical exercise causes a decrease in the recruitment of adhesion molecules, thus reducing the inflammatory process in the vascular wall [20]. Speretta [21] found that physical exercise positively affected the inflammation parameters, body weight, adipocyte area, and lipid profile in obese rats, but the influence of the different types of exercise gave different results on the analyzed parameters.

Aerobic exercise training is a well-established means for improving the vascular health of individuals with endothelial dysfunction. Most studies have shown that high-intensity interval training (HIIT) improves endothelial function more than moderate-intensity continuous training (MICT). Consistent with these findings, HIIT is reportedly more effective than MICT in increasing antioxidant status and NO availability. HIIT generates better blood flow than MICT due to the large shear stress it induces, and shear stress induces eNOS to produce NO to protect against oxidative stress [22]. How HIIT and MICT differ in affecting endothelial function in the hyperglycemic condition through the increase of GLP-1 release remains elusive.

The main purpose of this study was to compare and determine how HIIT and MICT may prevent endothelial dysfunction under hyperglycemic conditions. We compared the effects of both types of exercise training on GLP-1 level, RAGE gene expression, eNOS level, malondialdehyde (MDA) level, SOD activity, TNF*α* level, NF-*κβ* gene expression, and the histological appearance of aortic tissue from hyperglycemic rats.

## 2. Materials and Methods

This is an experimental study without blinding. Analysis was not performed by investigators blinded to the allocation of animals. This research was approved by the Health Research Ethics Committee of FKUI (number 1018/UN2.F1/ETIK/2017).

### 2.1. Animals

Twenty-four male Wistar rats (*Rattus norvegicus*), aged eight weeks and weighing 180–300 g, were used in this study. The rats were given standard food and drink ad libitum. The cages were kept clean and set to 12 h of light and 12 h of darkness. The ambient temperature was maintained at 23.1°C. Before the experiment, the rats were adjusted to the research environment for seven days. The rats were randomly divided into four groups: the control group with no intervention (C), the hyperglycemic group without intervention (HG-C), the hyperglycemic group that underwent the HIIT program (HG-IT), and the hyperglycemic group that underwent the MICT program (HG-CT). The rats in all the groups, except the C group, were injected intraperitoneal with a single dose of 40 mg/kg streptozotocin (STZ) (Sigma-Aldrich, Saint Louis, MO) dissolved in 0.1 M citrate buffer (pH 4.5) to induce hyperglycemia. Blood glucose was measured 72 h after injection, and blood was taken from the tail vein. Rats with a blood glucose level >200 mg/dL were considered hyperglycemic. All rats developed hyperglycemia 72 h after injection.

### 2.2. Exercise Procedures

**Figure 1:**
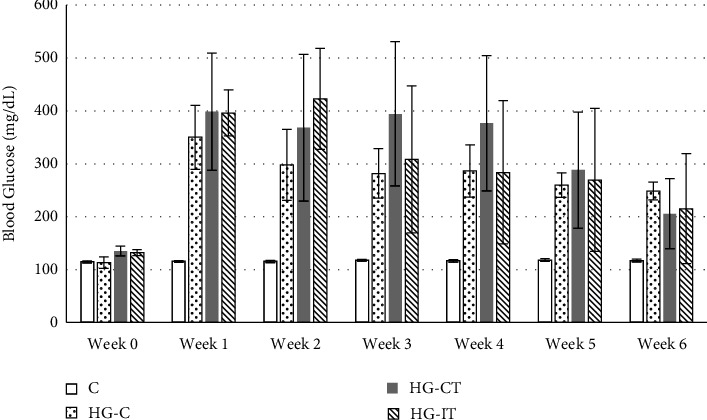
The weekly blood glucose levels (mg/dL). c: control group; HG-C:  hyperglycemic control group; HG-CT:  hyperglycemic rats that performed moderate-intensity continuous training (MICT); HG-IT:  hyperglycemic rats that performed high-intensity interval training (HIIT).

Physical exercise was performed six days a week, on a treadmill, with increasing speed, duration, and intensity each day. The MICT started at a speed of 27 m/min for 25 min, and the duration was increased every day (Table 1). The HIIT regimen was divided into odd and even days. On odd days, the training started at a speed of 40 m/min, with two repetitions (reps) of 3 min interspersed with 1 min of active rest (total duration = 7 min). The speed, reps, and duration were increased every subsequent odd day. On even days, the training started at a speed of 54 m/min, with three reps of 30 s interspersed with 1 min of active rest (total duration = 3.5 min). The speed, reps, and duration were increased every subsequent even day (Table 2). In both the MICT and HIIT, the training regimen started with a warm-up session (5 min at 16 m/min) and ended with a cool-down session (5 min at 16 m/min) (Tables 1 and 2). The exercise prescriptions were adapted from Afzalpour et al. [23]. Exercise intensity was confirmed using a treadmill speed reference for eight-week-old untrained Wistar rats from Qin et al. [24]. Blood sugar was evaluated each week during the provision of physical exercise (Figure 1).

### 2.3. Tissue Collection

The day after the exercise regimen was completed, decapitation was performed. The rats underwent glucose loading (2.5 g glucose/kg) for 30 min before surgery to induce GLP-1 release. Total anesthesia was conducted by intraperitoneal injection of combined xylazine hydrochloride (0.01 ml/kg) and ketamine (0.05 ml/kg). The ascending aorta until the abdominal aorta was surgically removed. Aortic tissue was rinsed in phosphate buffer saline (PBS) (pH 7.4) to remove excess blood and stored at −80°C.

On the day of measurement, the tissue was thawed at 2–8°C and was chopped in PBS with a glass homogenizer on ice. The homogenate was centrifuged at 2000–3000 RPM for 20 minutes and the supernatant was taken for measurement.

### 2.4. Enzyme-Linked Immunosorbent Assay

The concentrations of GLP-1 and eNOS were measured using ELISA kits from Bioenzy (BZ-08189170-EB and BZ-08185640-EB) and the concentration of TNF*α* was measured using ELISA kits from Cusabio (CSB-E11987 r) according to the manufacturer's instructions. Total protein was measured using the Bradford assay for normalization to GLP-1 and TNF*α* (Bio-Rad Laboratories).

### 2.5. Colorimetric Assay

SOD activity was measured using a colorimetric assay kit from Elabscience (E-BC-K020-M). The measurement was carried out using the water-soluble tetrazolium salts-1(WST-1) principal method. MDA levels were assessed using a colorimetric assay kit from Elabscience (E-BC-K025-S) and a modified spectrophotometric thiobarbituric acid (TBA) test method.

### 2.6. Quantitative Real-Time PCR

Total RNA was extracted from aortic umbilical tissue using the Quick-RNA MiniPrep Plus kit (Zymo Research) and was reverse transcribed using the ReverTra ACE® qPCR RT Master Mix with gDNA remover (Toyobo) cDNA synthesis kit, according to the manufacturer's instructions. A real-time polymerase chain reaction (RT-PCR) was performed using the SensiFAST SYBR Hi-Rox Kit (Bioline). The thermal cycling program was 95°C for 15 min followed by 40 cycles at 95°C for 20 s, 58°C for 30 s, and then 72°C for 30 s. The RAGE gene was detected, and glyceraldehyde 3-phosphate dehydrogenase (GAPDH) was used as the internal control or for normalization. The gene primer sequences used were as follows: RAGE: forward 5′-CAGAAACCGGTGATGAAGGAC-3′ and reverse 5′-TCTGGGTTGTCGTTTCGC-3′; NF-*κ*B: forward 5′-CGACGTATTGCTGTGCCTTC-3′ and reverse 5′-TTGAGATCTGCCCAGGTGGTA-3′; and GAPDH: forward 5′-GCATCTTCTTGTGCAGTGCC-3′ and reverse 5'-TACGGCCAAATCCGTTCACA-3′. The 2ΔΔct method was used to evaluate the relative expression in the control and treatment groups.

### 2.7. Hematoxylin and Eosin (H&E) Staining

The tissue samples from the ascending aorta until the abdominal aorta of the rats in all study groups were fixed with optimal cutting temperature (OCT) medium. They were then cut using a cryostat (Leica Model CM1950) with 4.5 um–5 um thick metal grids and brushes. They were stained using hematoxylin-eosin solution (0.1% Mayers hematoxylin) (Sigma; MHS-16).

The image of the stained preparation was taken using a Leica microscope and the Leica Application Suite software. The images were analyzed using the image J image processing program. The thickness of the aorta was measured starting from the tunica intima to the media. It was measured from four fields of view in a clockwise direction (12, 3, 6, and 9) and viewed with x400 magnification. The diameter of the aorta was viewed by x40 magnification and measured in the largest and smallest part; the results were added and then divided by two.

### 2.8. Statistical Analysis

Data are presented as mean ± standard deviation (SD) for normally distributed data and as mean (interquartile range) if the data are not normally distributed. Data normality was assessed using the one-sample Kolmogorov–Smirnov test. Statistical differences between multiple groups of data were assessed by one-way analysis of variance (ANOVA) followed by Tukey's post hoc test (normally distributed data) or the Kruskal–Wallis test followed by the Mann–Whitney *U* test (nonnormally distributed data). Statistical significance was defined as *p* < 0.05. All analyses were accomplished using Statistical Package for Social Sciences (SPSS) statistical software version 25 (SPSS Inc., IBM, USA).

## 3. Results

### 3.1. Blood Glucose

All rats had normal blood glucose levels before the start of the study. Except for the control group that did not get streptozotocin injection, blood glucose levels were always maintained above 200 mg/dL during the study. Although still considered hyperglycemia, the mean blood glucose level in the exercised group (both MICT and HIIT) tends to decline each week. On the contrary, the hyperglycemia group that did not receive exercise showed similar blood glucose levels in each week (Figure 1). Statistical analysis using two-way ANOVA showed a significant difference in mean blood glucose levels between the control group and all other groups all week, except Week 0 (*p* < 0.01). There was no significant difference in blood glucose levels between the HG-C, HG-IT, and HG-CT groups each week (*p* > 0.01).

### 3.2. Enzyme-Linked Immunosorbent Assay

**Figure 2:**
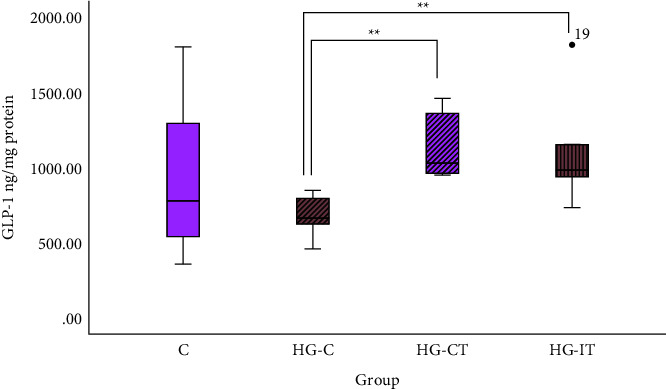
GLP-1 concentration within each group. c: control group; HG-C :  hyperglycemic control group; HG-CT:  hyperglycemic rats that did moderate-intensity continuous training (MICT); HG-IT:  hyperglycemic rats that did high-intensity interval training (HIIT). The error bars represent the standard deviation of measurements. $\begin{array}{c} {\;}^{**}p<0.01\text{.} \end{array}$

As shown in Figure 2, GLP-1 levels in the aortic tissues of the HG-C group (674.54 ± 134.97 ng/mg protein) were significantly lower than those in the aortic tissues of the HG-CT group (1,129.78 ± 224 ng/mg protein; *p* = 0.002) and the HG-IT group (91,099.03 ± 375.9 ng/mg protein; *p* = 0.009). There was no significant difference between the C group and both intervention groups (HG-CT and HG-IT). There was no significant difference in GLP-1 levels between the HG-CT and HG-IT groups.

**Figure 3:**
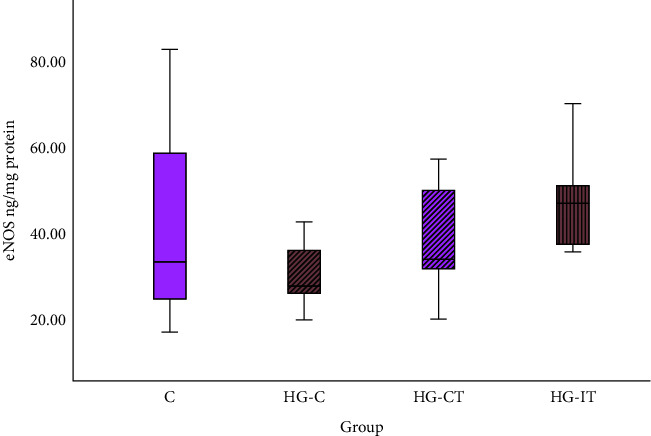
eNOS concentration within each group. c: control group; HG-C:  hyperglycemic control group; HG-CT :  hyperglycemic rats that performed moderate-intensity continuous training (MICT); HG-IT:  hyperglycemic rats that performed high-intensity interval training (HIIT). The error bars represent the standard deviation of measurements.

Figure 3 illustrates that there was no statistical difference in eNOS levels within each group. The mean eNOS level in the HG-IT group was 47.915 ± 12.5 ng/mg protein and 37.653 ± 13.259 ng/mg protein in the HG-CT group.

**Figure 4:**
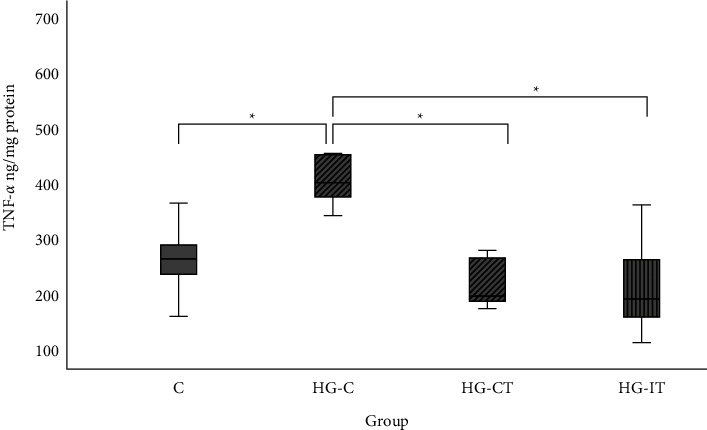
TNF*α* concentration within each group: c: control group; HG-C:  hyperglycemic control group; HG-CT:  hyperglycemic rats that performed moderate-intensity continuous training (MICT); HG-IT:  hyperglycemic rats that performed high-intensity interval training (HIIT). The error bars represent the standard deviation of measurements. ^*∗*^*p* < 0.05.

Figure 4 shows that the TNF*α* concentration in the HG-C group (444 ± 124.56 ng/mg protein) was significantly higher (*p* = 0.001) than in the HG-CT group (218 ± 44.22 ng/mg protein) and the HG-IT group (215 ± 89.121 ng/mg protein). There was no significant difference in TNF*α* levels between the HG-CT and HG-IT groups.

### 3.3. Colorimetric Assay

**Figure 5:**
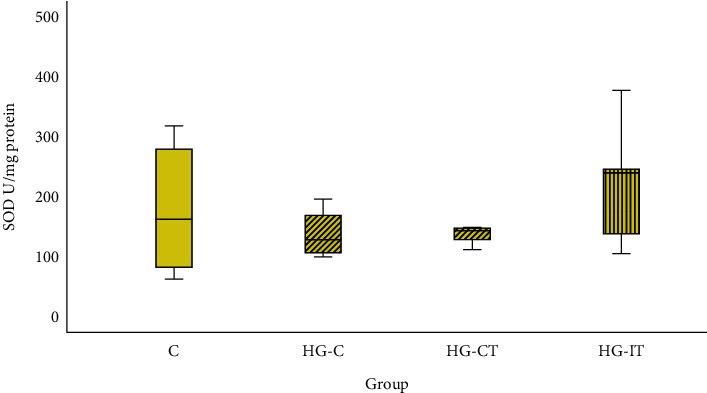
SOD activity within each group. c: control group; HG-C:  hyperglycemic control group; HG-CT:  hyperglycemic rats that performed moderate-intensity continuous training (MICT); HG-IT:  hyperglycemic rats that performed high-intensity interval training (HIIT). The error bars represent the standard deviation of measurements.

As shown in Figure 5, there was no significant difference in the SOD activity within all groups.

**Figure 6:**
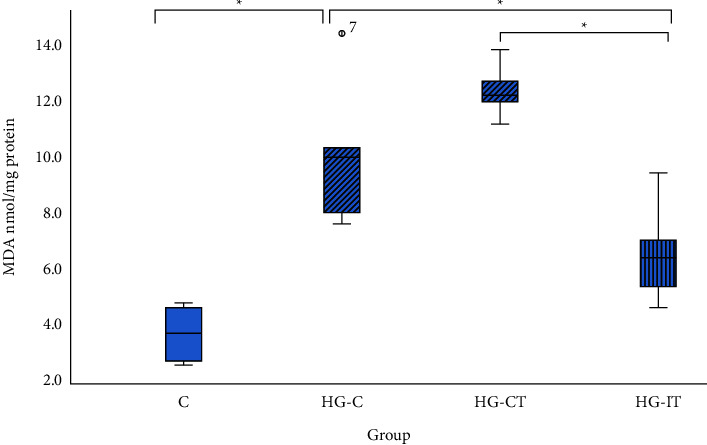
MDA concentration within each group. c: control group; HG-C:  hyperglycemic control group; HG-CT:  hyperglycemic rats that performed moderate-intensity continuous training (MICT); HG-IT:  hyperglycemic rats that performed high-intensity interval training (HIIT). The error bars represent the standard deviation of measurements. ^*∗*^*p* < 0.05.

In terms of MDA levels, the C group had a significantly lower MDA level (3.65 ± 0.92 nmol/mgprotein) when compared to the HG-C group (*p* < 0.05) (Figure 6). The MDA level in the HG-IT group (6.5 ± 1.67 nmol/mgprotein) was significantly (*p* = 0.006) lower than that in the HG-C group (10 ± 2.4 nmol/mgprotein). However, there was no significant difference in MDA levels between the HG-C and HG-CT groups.

### 3.4. Quantitative Real-Time PCR

**Figure 7:**
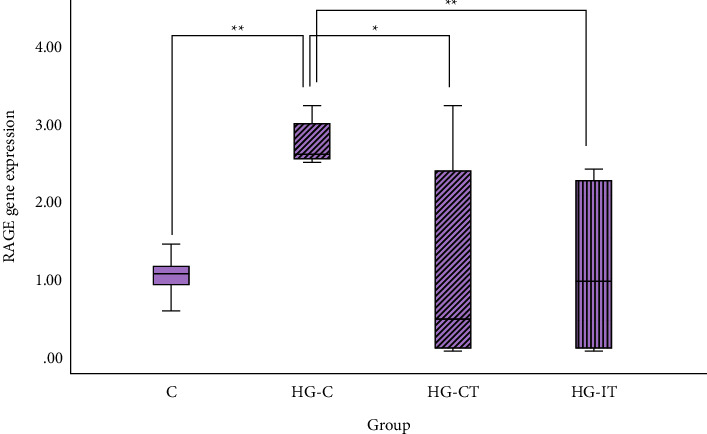
RAGE gene expression within each group. c: control group; HG-C:  hyperglycemic control group; HG-CT:  hyperglycemic rats that performed moderate-intensity continuous training (MICT); HG-IT:  hyperglycemic rats that performed high-intensity interval training (HIIT). The error bars represent the standard deviation of measurements.  ^*∗∗*^*p* < 0.01; ^*∗*^*p* < 0.05.

Figure 7 shows the level of RAGE gene expression in the abdominal aorta tissue samples. RAGE gene expression was significantly higher in the HG-C group (2.74 ± 0.3) compared to the C group (1.004 ± 0.28; *p* = 0.002), the HG-CT group (1.2 ± 1.34; *p* = 0.041), and the HG-IT group (1.125 ± 1.09; *p* = 0.002). There was no significant difference in RAGE gene expression between the HG-CT and HG-IT groups.

**Figure 8:**
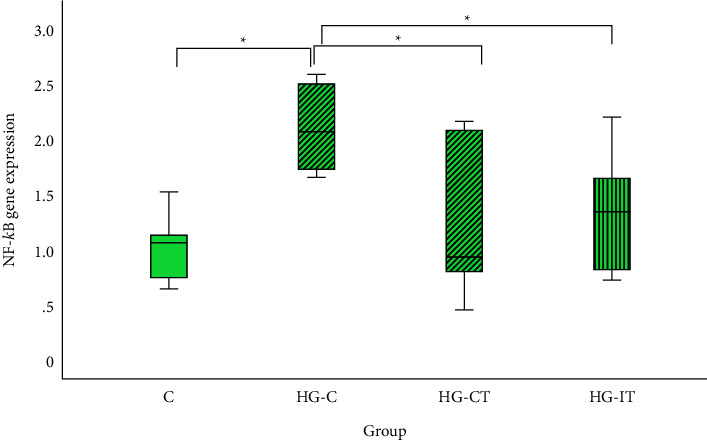
NF-*κ*B gene expression within each group. c: control group; HG-C:  hyperglycemic control group; HG-CT:  hyperglycemic rats that performed moderate-intensity continuous training (MICT); HG-IT:  hyperglycemic rats that performed high-intensity interval training (HIIT). The error bars represent the standard deviation of measurements. ^*∗*^*p* < 0.05.

As shown in Figure 8, the HG-C group (2.1 ± 0.4) had the highest NF-*κ*B gene expression and was significantly higher than that in the C group (1.03 ± 0.31; *p* = 0.004), the HG-CT group (1.2 ± 0.71; *p* = 0.05), and the HG-IT group (1.4 ± 0.57; *p* = 0.016). There was no significant difference in NF-*κ*B gene expression between the HG-CT and HG-IT groups.

**Figure 9:**
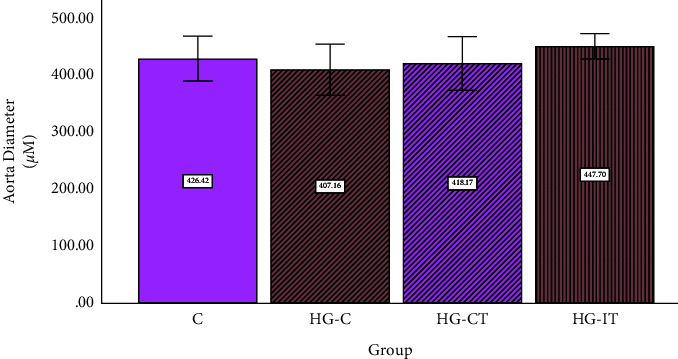
Aorta diameter within each group. c: control group; HG-C:  hyperglycemic control group; HG-CT:  hyperglycemic rats that performed moderate-intensity continuous training (MICT); HG-IT:  hyperglycemic rats that performed high-intensity interval training (HIIT). The error bars represent the standard deviation of measurements.

**Figure 10:**
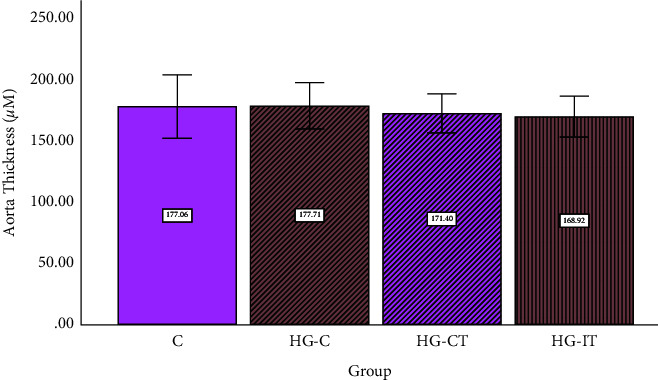
Aortic wall thickness within each group. c: control group; HG-C:  hyperglycemic control group; HG-CT:  hyperglycemic rats that performed moderate-intensity continuous training (MICT); HG-IT:  hyperglycemic rats that performed high-intensity interval training (HIIT). The error bars represent the standard deviation of measurements.

**Figure 11:**
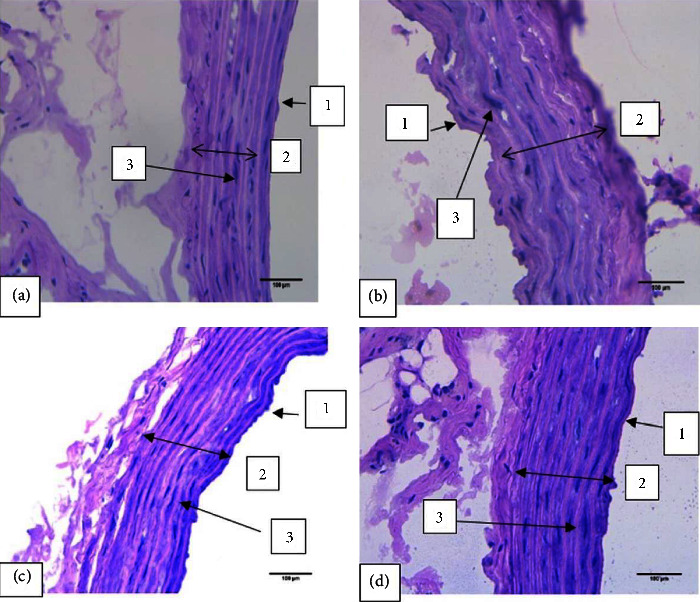
Microscopic view of the aortic wall stained with hematoxylin-eosin at 400× magnification. Tunica intima (endothelial) detachment and an irregularity in the arrangement of collagen fibers and smooth muscle cells in the tunica media were observed in the hyperglycemic control group. (a) Control group. (b) Hyperglycemic control group. (c) Hyperglycemic rats that performed moderate-intensity continuous training (MICT). (d) Hyperglycemic rats that performed high-intensity interval training (HIIT). (1) Tunica intima. (2) Tunica media. (3) Smooth muscle cells (SMC).

**Figure 12:**
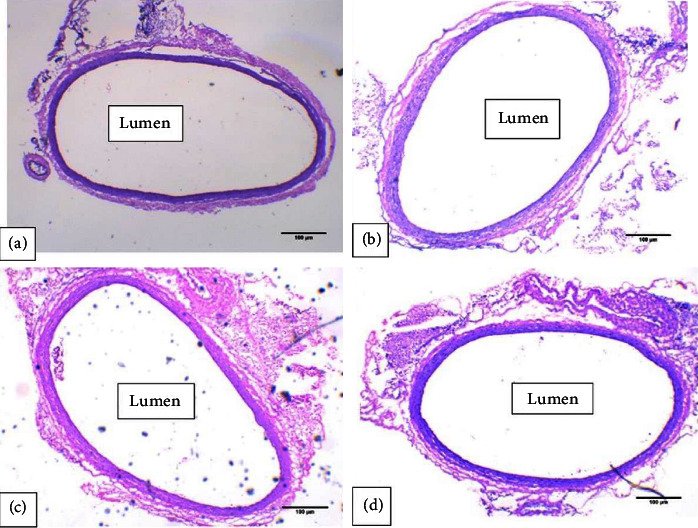
Microscopic view of the aortic lumen stained with hematoxylin-eosin at 40× magnification. An irregularity in the arrangement of the walls was observed in the hyperglycemic control group. (a) Control group. (b) Hyperglycemic control group. (c) Hyperglycemic rats that performed moderate-intensity continuous training (MICT). (d) Hyperglycemic rats that performed high-intensity interval training (HIIT).

It was found that there were no significant differences in the aorta diameter and wall thickness between the groups (Figures 9 and 10). However, when the tissues were examined using light microscopy, tunica intima (endothelial) detachment was observed in the HG-C group samples. An irregularity in the arrangement of collagen fibers and smooth muscle cells (SMCs) in the tunica media was also seen (Figures 11 and 12).

## 4. Discussion

Our study examined the direct effect of 6 weeks of exercise on the aorta of rats under hyperglycemic conditions. To the best of our knowledge, it is the first to explore the effect of exercise on the GLP-1 levels and how it then inhibits AGE-RAGE, hinders inflammation, reduces stress oxidative, and protects vascular structure and function. The main finding of this study is a higher GLP-1 concentration, lower RAGE gene expression, lower TNF*α* concentration, and lower NF-*κβ* gene expression in hyperglycemic rats that performed both HIIT and MICT compared to hyperglycemic control rats. It is worth noting that lower MDA concentrations were found only in HIIT rats, while no differences in SOD activity or eNOS levels were found across all groups.

GLP-1 is one of the incretin hormones that have multifaceted and broad pharmacological potential. It is activated by binding to its receptor, GLP-1R. GLP-1R is expressed in various organs in the body, including the cardiovascular system, and specifically in SMCs, the endothelium, and cardiomyocytes [13, 25]. According to the findings of Bhats et al. [26], GLP-1 level in rats with T2DM is lower compared to healthy rats. This is presumably due to several factors. The first factor is chronic hyperglycemia (>72 h). After oral consumption of glucose, the glucose level in the intestine increases, which induces L cells to increase GLP-1 secretion. However, in the case of chronic hyperglycemia, diabetic products are triggered, such as glycated serum (GS) and glucolipoxity [26, 27]. GS plays a role in reducing the expression of prohormone convertase 1/3 (PC 1/3), an enzyme that functions in the posttranslation process of converting proglucagon into GLP-1 in intestinal L cells [28]. The second factor is the presence of systemic inflammation caused by chronic hyperglycemia. Inflammatory environments induce the synthesis and release of IL-6. There have been reports of the synthesis of IL-6 by various cell types, including immune cells, endothelial cells, skeletal and smooth muscle cells, thyroid cells, fibroblasts, mesangial cells, keratinocytes, microglial cells, astrocytes, certain tumor cells, and islet cells. [29] In T2DM, adipocytes and adipose tissue macrophages are the major sources of increased circulating IL-6. These inflammatory cytokines may be circulated throughout the body, affecting organs and tissues further away, such as the kidneys, skeletal and heart muscle, and circulating leukocytes [30]. IL-6 that was synthesized and secreted during inflammatory conditions lead to sustained high ROS levels [31]. ROS interferes with insulin signaling, which leads to inhibition of glucose uptake and disruption of insulin secretion. Insulin is known to stimulate proglucagon gene expression and GLP-1 synthesis. Hence, disruption of insulin secretion results in GLP-1 deficiency. The final factor is the presence of dipeptidyl peptidase 4 (DPP-4). This enzyme can degrade the active form of GLP-1 (7–36) to a noninsulin tropic form of GLP-1 (9–36) that causes the active form of GLP-1 to last for only one to two minutes [25, 32]. In contrast to Bhat et al. who measured GLP-1 in animal serum immediately after the intervention (up to 1 hour), GLP-1 measurements in our study were performed on aortic tissue 24 hours after the intervention. We suspect it contributes to the lack of difference in GLP-1 levels between healthy and diabetic rats in our study.

Here, we have shown that rats performing HIIT and MICT had higher levels of GLP-1 than hyperglycemic control rats. This could be due to sympathetic activation provoked by exercise that induces modulation of GLP-1 release via an increase in epinephrine [33–35]. Epinephrine is known to stimulate the release of GLP-1 in the mouse ileum [36]. Ellingsgaard et al. also showed that physical exercise could increase GLP-1 levels through skeletal muscle contraction-induced interleukin 6 (IL-6) secretion. Muscle contraction by both type I and type II muscle fibers increases cytosolic calcium (Ca^2+^) and activates p38 mitogen-activated protein kinase (MAPK) and/or calcineurin, which can further activate transcription factors of genes that encode IL-6 in the nucleus of myocytes [37–39]. IL-6 is then released into the bloodstream and binds to receptors located on intestinal L cells [40, 41]. This binding increases the phosphorylation of signal transducer and transcription activator 3 (STAT3) via Janus kinase 2 (JAK 2), which triggers an increase in Ca^2+^ levels, thereby inducing GLP-exocytosis [41]. IL-6 can also induce GLP-1 synthesis by increasing proglucagon transcription and PC1/3 expression in intestinal L cells [41].

The result of our study shows that under hyperglycemic condition, RAGE levels are lower in rats that performed both HIIT and MICT compared to hyperglycemic control rats. Physical exercise indirectly inhibits AGEs-RAGE activation via the GLP-1 signaling pathway. Tang et al. showed that GLP-1 could inhibit RAGE expression [13]. When GLP-1 binds to GLP-1R, it is activated and this increases the cAMP level, which activates the protein kinase A (PKA) signaling pathway. The cAMP pathway suppresses RAGE gene expression and reduces AGEs-RAGE activation [13]. In addition, Lim et al. showed that under hyperglycemic conditions, GLP-1 could increase eNOS mRNA expression via the cAMP signaling pathway that blocks the AGEs-RAGE pathway [42]. Therefore, it is speculated that physical exercise, which stimulates an increase in GLP-1 secretion, increases eNOS mRNA expression [42]. According to a study by Farinha et al., high-intensity exercise training decreased sRAGE in insulin-dependent patients, which may be related to greater plasma total antioxidant capacity levels and enhanced antioxidant enzyme activity [43]. Exercise is thought to encourage a more effective utilization of reactive intermediates of glycolytic and polyol pathways, thus reducing their availability for reaction with amino groups and the formation of AGEs [44].

Under the hyperglycemic conditions associated with T2DM, AGEs increase rapidly and reduce the half-life of eNOS mRNA, which causes eNOS deficiency [45]. In addition, the AGEs-RAGE interaction in the formation of an extracellular matrix causes structural changes in the blood vessel walls, thereby worsening vascular conditions [45]. Changes in the structure of blood vessel walls disrupt the supply of nutrients and materials to various body organs because the blood vessels constrict, and NO is not released. The effects of these morphological changes can increase the risk of stroke, blindness, ischemia, retinopathy, atherosclerosis, heart attack, kidney failure, and gangrene of the lower extremities.

It is known that physical exercise has a positive impact on preventing vascular complications in DM through the inhibition of chronic inflammation. You et al. and Gleeson et al. reported several studies that explain the mechanism of how exercise training reduces chronic inflammation. Every bout of exercise causes contracting muscle tissue to produce muscle-derived anti-inflammatory “myokine.” Exercise also has effect on adipose tissue to reduce hypoxia and local adipose tissue inflammation. In the immune system, exercise lowers the number of proinflammation production. Specifically, on endothelial cells, exercise training reduces leukocyte adhesion and cytokine formation [20, 46]. A study by Pramaningtyas showed that physical exercise triggers an increase in the NO released by eNOS through shear stress [47]. The NO then diffuses into the vascular smooth muscle and activates soluble guanylate cyclase-cyclic guanosine monophosphate (sGC-cGMP) signaling to inhibit L-type Ca^2+^ channel located on vascular smooth muscle cell membranes and to induce a decrease in cytosolic Ca^2+^ so that relaxation can occur [48]. During physical exercise, blood flow increases. Blood pressure exerts radial compression and circular-attraction forces on various types of cells in the vessel wall and on extracellular matrix proteins. Blood flow also applies a longitudinal frictional force on the tunica intima of blood vessels and produces shear stress (velocity per unit area) [49]. In turn, shear stress promotes optimal regulation of eNOS. Chengji et al. showed that six weeks of physical exercise of different intensities could increase eNOS mRNA, eNOS activity, and levels of NO product (nitrite and nitrate). Increasing the intensity of physical exercise further increases eNOS levels. This is thought to be influenced by the speed of the shear stress induced by the intensity of the physical exercise [48]. A study by Grijalva et al. found significantly higher eNOS protein levels and eNOS dimerization in the myocardium of a diabetic rat model given exercise training compared to sedentary rats [50]. In this study, we failed to demonstrate differences in eNOS activity in the aorta tissue across the groups. We can only hypothesize that decapitation of the rat's 24-hour postexercise played a role in affecting the levels of eNOS measured in the aorta.

Our study results show no differences in eNOS levels between groups given HIIT and MICT. The activity of eNOS is affected by 2 pathways. The first pathway is the initiation of eNOS by shear stress. The speed of shear stress is higher with increased intensity of physical exercise and thus further increases eNOS levels [48]. Through this mechanism, higher intensity in HIIT compared to MICT results in greater shear stress. The second pathway is eNOS release through AGE-RAGE inhibition. Physical exercise triggers the release of IL-6 that causes an increase of GLP-1. Further increased level of GLP-1 inhibits AGE-RAGE. The increase in plasma IL-6 is more influenced by duration so that the intensity is adjusted to the duration [37].

In the hyperglycemic state, very high levels of lipid peroxidation and decreased antioxidant defense mechanisms can simultaneously cause damage to cellular organelles and create oxidative stress. In patients with T2DM, the increase in lipid peroxidation products (MDA) affects the decreasing antioxidant enzymes, such as glutathione (GSH) and SOD [51]. Our results are consistent with previous studies. The mean MDA level in the control group was significantly lower than that in the hyperglycemic control group. However, compared to the hyperglycemic control group, in this study, we found that the MDA level was only lower in HIIT and not MICT group. This is in line with the findings of Mitranun et al. They also compared MICT with HIIT and found that MDA levels in subjects that performed HIIT were significantly lower than the levels in subjects that performed MICT [52]. We assumed that the processes through which interval exercise reduces oxidative damage were more effective compared to continuous training. Interval aerobic training appears to have a stronger effect on blood flow and shear stress than continuous training, which in turn improved NO bioavailability and endothelium-dependent vasodilation. An earlier study by Liu et al. found that human mesenchymal stem cells respond better to intermittent fluid shear stress than continuous fluid shear stress to promote osteogenic differentiation [53]. Mitranun et al. showed increased plasma nitrite and nitrate concentrations in the interval group but not in the continuous group. Therefore, reduced oxidative stress may be associated with improved endothelial function following interval training [52]. It is also plausible that interval training exercise sessions caused higher shear stress and thus lead to greater cellular and molecular reactions [54].

According to a study by Lee et al., moderate-intensity physical exercise can increase SOD activity under hyperglycemic conditions due to decreased NADPH oxidase activity, thus inhibiting endothelial dysfunction [55]. NADPH oxidase is a membrane-bound protein with the main function to transfer electrons across the plasma membrane to molecular oxygen, which results in the generation of the superoxide anion and subsequently reactive oxygen species (ROS), including hydrogen peroxide (H_2_O_2_) and hydroxyl radicals (OH^●^). [56].

Sarasvati et al. demonstrated a significant increase in SOD activity in subjects who performed HIIT compared to subjects who performed MICT. It is assumed that MICT does not produce an adaptive response to oxidative stress via activation of the nuclear erythroid 2-related factor (Nrf2) signal. Nrf2 is a redox transcription factor and a major regulator of antioxidants [57]. The higher SOD activity in subjects who performed HIIT was due to the activation of the redox protein sensitivity pathway via higher ROS production [58]. HIIT could provoke greater blood flow in the large blood vessels during exercise in which shear stress increases and induces NO bioavailability [57]. We did not find any significant differences in SOD activity among the groups, which is in contrast to the findings of Sarasvati et al. However, our result is in line with the study result of Kesavulu et al. which showed an increased erythrocyte catalase (CAT) activity and decreased glutathione peroxidase (GPx) activity in diabetic patients compared to controls, but there was no significant change in superoxide dismutase (SOD) activity [59].

Nuclear transcription factor *κ*B (NF-*κ*B) is activated by AGEs and in turn promotes the transcription of inflammatory markers, which produces vasoconstrictor molecules and oxidative damage [60]. In our study, NF-*κ*B gene expression in the hyperglycemic control group was significantly higher compared to the expression in the control group. This result is in line with previous studies that found hyperglycemia can produce oxidative stress in the endothelium that activates NF-*κ*B [61, 62]. Recent studies show that NF-*κ*B is the main suspect and a key player in the development of insulin resistance and T2DM [61]. TNF*α*, a regulated product of NF-*κ*B and a strong activator of NF-*κ*B, induces insulin resistance mainly through serine phosphorylation of insulin receptor substrate-1 (IRS1) [61]. Research by Patel et al. has shown that long-standing hyperglycemia will generate AGEs and directly activate NF-*κ*B in vascular smooth muscle cells (VSMCs) [61]. Under hyperglycemic conditions, VSMCs showed a higher basal NF-*κ*B activity than under normoglycemic conditions [61].

Previous studies have stated that undertaking physical activity can reduce NF-*κ*B expression through the IL-10 pathway. Anti-inflammatory IL-10 inhibits TNF*α* by suppressing NF-*κ*B P65 expression in macrophages [63]. This is in accordance with the result of our study which showed significantly lower NF-*κ*B expression in animals that performed both HIIT and MICT compared to animals in the hyperglycemic control group. Kahkha et al. reported that serum NF-*κ*B levels were higher in subjects who performed HIIT than in those who performed aerobic exercise [64]. However, there was no significant difference between the two training methods found in our study result.

In this study, we examined the effect of HIIT and MICT on inflammation by analyzing TNF*α* levels. TNF*α* is a potent proinflammatory agent that regulates many aspects of macrophage function. It is rapidly released after trauma or bacterial infection, and it is one of the most abundant early mediators in inflammation [65]. Our results showed that the expression of TNF*α* was significantly higher in the hyperglycemic control group than in the exercising group. This is in accordance with previous studies that have shown that exercising at a certain intensity can provide positive benefits in terms of reducing inflammatory factors [66]. Over the past two decades, it has become increasingly clear that chronic inflammation is an important contributing factor in the development of chronic noncommunicable diseases, such as T2DM. The inflammatory process partly mediates vascular complications in patients with diabetes, and there is evidence that IL-1*β* and TNF*α* are the major proinflammatory mediators in cell damage and insulin resistance [66]. During exercise, skeletal muscle cells contract and thus activate c-JunN-terminal kinase (JNK/AP-1) and MAPK, which stimulate IL-1ra and IL-10, thereby inhibiting the production of TNF*α* [67, 68]. Our results indicate that both HIIT and MICT have a positive effect in that they reduce TNF*α* under hyperglycemic conditions.

In chronic hyperglycemia, the aorta is at risk of endothelial dysfunction. Endothelial dysfunction is a condition that can be initiated through the activation of AGEs-RAGE [13]. It can affect the histology of the aorta, especially the thickness of the intima-media and the lumen diameter. However, in this study, there was no difference in the aorta diameter and the thickness of tunica intima-media across all the groups. It is assumed that six weeks is not a sufficient length of time for significant hyperglycemia-related histological changes to occur in the aorta. Nevertheless, we found that the tunica intima in the hyperglycemic control group was detached (endothelial detachment). This means that the aorta was not evenly enveloped, as shown in Figure 11. In addition, there was an irregularity in the arrangement of the collagen fibers and SMCs of the tunica media, a characteristic of endothelial dysfunction. This is thought to be related to apoptosis induced by hyperglycemia. Furthermore, the increase in AGEs can cause changes in endothelial cell structure through AGE-RAGE activation. Changes in the structure of endothelial cells increase vascular permeability, which is followed by loss of adherence junctions, contraction of SMCs, and focal adhesion redistribution. Increased permeability results in protein leakage into the tunica media. Moreover, SMC contraction has a vital role in the regulation of endothelial permeability. The actin-myosin interaction is regulated by myosin light chain (MLC) phosphorylation, which causes changes in the distribution of F-actin and barrier failure, resulting in irregularities in the tunica media. Optimal control of MLC is mediated by a balance between myosin light chain phosphatase (MLCP) and myosin light chain kinase (MLCK) [13].

## 5. Conclusions

In this study, hyperglycemic rats that performed HIIT and MICT had significantly higher GLP-1 levels, lower RAGE gene expression, lower TNF*α* levels, and lower NF-*κ*B gene expression than hyperglycemic rats that had no intervention. Microscopic examination of aortic tissue revealed that hyperglycemic rats that underwent the exercise treatment had better tissue arrangement than hyperglycemic rats that did not undergo the exercise treatment. Except for the MDA levels, it can be concluded that, in the presence of hyperglycemia, HIIT and MICT have similar protective effects against endothelial dysfunction. HIIT has the advantage of being less time-consuming than MICT.

## Figures and Tables

**Table 1:** The continuous training protocol [23].

Training week	Days	Warming up	Duration (min)	Intensity	Cooling down
Week 1	1	5 min (16 m/min)	30	27 m/min	5 min (16 m/min)
	2	5 min (16 m/min)	32	27 m/min	5 min (16 m/min)
	3	5 min (16 m/min)	34	27 m/min	5 min (16 m/min)
	4	5 min (16 m/min)	36	27 m/min	5 min (16 m/min)
	5	5 min (16 m/min)	38	27 m/min	5 min (16 m/min)
	6	5 min (16 m/min)	40	27 m/min	5 min (16 m/min)

Week 2	1	5 min (16 m/min)	42	27 m/min	5 min (16 m/min)
	2	5 min (16 m/min)	44	27 m/min	5 min (16 m/min)
	3	5 min (16 m/min)	46	27 m/min	5 min (16 m/min)
	4	5 min (16 m/min)	48	27 m/min	5 min (16 m/min)
	5	5 min (16 m/min)	50	27 m/min	5 min (16 m/min)
	6	5 min (16 m/min)	52	27 m/min	5 min (16 m/min)

Week 3	1	5 min (16 m/min)	54	27 m/min	5 min (16 m/min)
	2	5 min (16 m/min)	56	27 m/min	5 min (16 m/min)
	3	5 min (16 m/min)	58	27 m/min	5 min (16 m/min)
	4	5 min (16 m/min)	60	27 m/min	5 min (16 m/min)
	5	5 min (16 m/min)	62	27 m/min	5 min (16 m/min)
	6	5 min (16 m/min)	64	27 m/min	5 min (16 m/min)

Week 4	1	5 min (16 m/min)	66	27 m/min	5 min (16 m/min)
	2	5 min (16 m/min)	68	27 m/min	5 min (16 m/min)
	3	5 min (16 m/min)	70	27 m/min	5 min (16 m/min)
	4	5 min (16 m/min)	70	27 m/min	5 min (16 m/min)
	5	5 min (16 m/min)	70	27 m/min	5 min (16 m/min)
	6	5 min (16 m/min)	70	27 m/min	5 min (16 m/min)

Weeks 5–6	1	5 min (16 m/min)	70	27 m/min	5 min (16 m/min)
	2	5 min (16 m/min)	70	27 m/min	5 min (16 m/min)
	3	5 min (16 m/min)	70	27 m/min	5 min (16 m/min)
	4	5 min (16 m/min)	70	27 m/min	5 min (16 m/min)
	5	5 min (16 m/min)	70	27 m/min	5 min (16 m/min)
	6	5 min (16 m/min)	70	27 m/min	5 min (16 m/min)

**Table 2:** The interval training protocol [23].

Training week	Days	Warming up	Duration	Intensity	Cooling down
Week 1	1	5 min (16 m/min)	17 min	2 rep. 3 min. 40 m/min. Active rest 13 m/min. 1 min.	5 min (16 m/min)
	2	5 min (16 m/min)	13,5 min	3 rep. 30 sec. 54 m/min. Active rest 16 m/min. 1 min.	5 min (16 m/min)
	3	5 min (16 m/min)	17 min	2 rep. 3 min. 40 m/min. Active rest 16 m/min. 1 min.	5 min (16 m/min)
	4	5 min (16 m/min)	16,5 min	5 rep. 30 sec. 54 m/min. Active rest 16 m/min. 1 min.	5 min (16 m/min)
	5	5 min (16 m/min)	17 min	2 rep. 3 min. 40 m/min. Active rest 16 m/min. 1 min.	5 min (16 m/min)
	6	5 min (16 m/min)	19,5 min	7 rep. 30 sec. 54 m/min. Active rest 16 m/min. 1 min.	5 min (16 m/min)
Week 2	1	5 min (16 m/min)	21 min	3 rep. 3 min. 40 m/min. Active rest 16 m/min. 1 min.	5 min (16 m/min)
	2	5 min (16 m/min)	22,5 min	9 rep. 30 sec. 54 m/min. Active rest 16 m/min. 1 min.	5 min (16 m/min)
	3	5 min (16 m/min)	21 min	3 rep. 3 min. 40 m/min. Active rest 16 m/min. 1 min.	5 min (16 m/min)
	4	5 min (16 m/min)	25,5 min	11 rep. 30 sec. 54 m/min. Active rest 16 m/min. 1 min.	5 min (16 m/min)
	5	5 min (16 m/min)	21 min	3 rep. 3 min. 40 m/min. Active rest 16 m/min. 1 min.	5 min (16 m/min)
	6	5 min (16 m/min)	28,5 min	13 rep. 30 sec. 54 m/min. Active rest 16 m/min. 1 min.	5 min (16 m/min)

Week 3	1	5 min (16 m/min)	25 min	4 rep. 3 min. 40 m/min. Active rest 16 m/min. 1 min.	5 min (16 m/min)
	2	5 min (16 m/min)	31,5 min	15 rep. 30 sec. 54 m/min. Active rest 16 m/min. 1 min.	5 min (16 m/min)
	3	5 min (16 m/min)	25 min	4 rep. 3 min. 40 m/min. Active rest 16 m/min. 1 min	5 min (16 m/min)
	4	5 min (16 m/min)	34,5 min	17 rep. 30 sec. 54 m/min. Active rest 16 m/min. 1 min.	5 min (16 m/min)
	5	5 min (16 m/min)	29 min	5 rep. 3 min. 40 m/min. Active rest 16 m/min. 1 min.	5 min (16 m/min)
	6	5 min (16 m/min)	37,5 min	19 rep. 30 sec. 54 m/min. Active rest 16 m/min. 1 min.	5 min (16 m/min)
Week 4	1	5 min (16 m/min)	29 min	5 rep. 3 min. 40 m/min. Active rest 16 m/min. 1 min.	5 min (16 m/min)
	2	5 min (16 m/min)	37,5 min	19 rep. 30 sec. 54 m/min. Active rest 16 m/min. 1 min.	5 min (16 m/min)
	3	5 min (16 m/min)	33 min	6 rep. 3 min. 40 m/min. Active rest 16 m/min. 1 min.	5 min (16 m/min)
	4	5 min (16 m/min)	39 min	20 rep. 30 sec. 54 m/min. Active rest 16 m/min. 1 min.	5 min (16 m/min)
	5	5 min (16 m/min)	33 min	6 rep. 3 min. 40 m/min. Active rest 16 m/min. 1 min.	5 min (16 m/min)
	6	5 min (16 m/min)	39 min	20 rep. 30 sec. 54 m/min. Active rest 16 m/min. 1 min	5 min (16 m/min)

Weeks 5–6	1	5 min (16 m/min)	33 min	6 rep. 3 min. 40 m/min. Active rest 16 m/min. 1 min.	5 min (16 m/min)
	2	5 min (16 m/min)	39 min	20 rep. 30 sec. 54 m/min. Active rest 16 m/min. 1 min.	5 min (16 m/min)
	3	5 min (16 m/min)	33 min	6 rep. 3 min. 40 m/min. Active rest 16 m/min. 1 min.	5 min (16 m/min)
	4	5 min (16 m/min)	39 min	20 rep. 30 sec. 54 m/min. Active rest 16 m/min. 1 min.	5 min (16 m/min)
	5	5 min (16 m/min)	33 min	6 rep. 3 min. 40 m/min. Active rest 16 m/min. 1 min.	5 min (16 m/min)
	6	5 min (16 m/min)	39 min	20 rep. 30 sec. 54 m/min. Active rest 16 m/min. 1 min.	5 min (16 m/min)

## Data Availability

The datasets generated and/or analyzed in the current study are available from the corresponding author upon reasonable request.

## References

[1] Oguntibeju O. O. (2019). Type 2 diabetes mellitus, oxidative stress and inflammation: examining the links. *International Journal of Physiology, Pathophysiology*.

[2] (2021). IDF Diabetes Atlas. https://diabetesatlas.org.

[3] Forbes J. M., Fotheringham A. K. (2017). Vascular complications in diabetes: old messages, new thoughts. *Diabetologia*.

[4] Maruhashi T., Higashi Y. (2021). Pathophysiological association between diabetes mellitus and endothelial dysfunction. *Antioxidants*.

[5] Maresch C. C., Stute D. C., Fleming T., Lin J., Hammes H. P., Linn T. (2019). Hyperglycemia induces spermatogenic disruption via major pathways of diabetes pathogenesis. *Scientific Reports*.

[6] Luc K., Schramm-Luc A., Guzik T. J., Mikolajczyk T. P. (2019). Oxidative stress and inflammatory markers in prediabetes and diabetes. *Journal of Physiology & Pharmacology*.

[7] Chen Q., Wang Q., Zhu J., Xiao Q., Zhang L. (2018). Reactive oxygen species: key regulators in vascular health and diseases. *British Journal of Pharmacology*.

[8] Lawrence T. (2009). The nuclear factor NF- B pathway in inflammation. *Cold Spring Harbor Perspectives in Biology*.

[9] Casella S., Bielli A., Mauriello A., Orlandi A. (2015). Molecular pathways regulating macrovascular pathology and vascular smooth muscle cells phenotype in type 2 diabetes. *International Journal of Molecular Sciences*.

[10] Zinatizadeh M. R., Schock B., Chalbatani G. M., Zarandi P. K., Jalali S. A., Miri S. R. (2021). The Nuclear Factor Kappa B (NF-kB) signaling in cancer development and immune diseases. *Genes & Diseases*.

[11] Taleb S. (2016). Inflammation in atherosclerosis. *Archives of Cardiovascular Diseases*.

[12] Voloshyna I., Littlefield M. J., Reiss A. B. (2014). Atherosclerosis and interferon-*γ*: new insights and therapeutic targets. *Trends in Cardiovascular Medicine*.

[13] Tang S. T., Tang Hq, Su H. (2019). Glucagon-likepeptide-1 attenuates endothelial barrier injury in diabetes via cAMP/PKA mediated down-regulation of MLC phosphorylation. *Biomedicine & Pharmacotherapy*.

[14] Tang S. T., Zhang Q., Tang Hq (2016). Effects of glucagon-likepeptide-1 on advanced glycation endproduct-induced aortic endothelial dysfunction in streptozotocin-induced diabetic rats: possible roles of Rho kinase- and AMP kinase-mediated nuclear factor *κ*B signaling pathways. *Endocrine*.

[15] Yamagishi S. i., Fukami K., Matsui T. (2015). Crosstalk between advanced glycation end products (AGEs)-receptor RAGE axis and dipeptidyl peptidase-4-incretin system in diabetic vascular complications. *Cardiovascular Diabetology*.

[16] Li J., Zheng J., Wang S., Lau H. K., Fathi A., Wang Q. (2017). Cardiovascular benefits of native GLP-1 and its metabolites: an indicator for GLP-1-therapy strategies. *Frontiers in Physiology*.

[17] Mohammad P., Esfandiar K. Z., Abbas S., Ahoora R. (2019). Effects of moderate-intensity continuous training and high-intensity interval training on serum levels of Resistin, Chemerin and liver enzymes in Streptozotocin-Nicotinamide induced Type-2 diabetic rats. *Journal of Diabetes and Metabolic Disorders*.

[18] Gibala M. J., Little J. P., MacDonald M. J., Hawley J. A. (2012). Physiological adaptations to low-volume, high-intensity interval training in health and disease. *The Journal of Physiology*.

[19] Kwak S. E., Lee J. H., Zhang D., Song W. (2018). Angiogenesis: focusing on the effects of exercise in aging and cancer. *Journal of Exercise Nutrition & Biochemistry*.

[20] You T., Arsenis N. C., Disanzo B. L., LaMonte M. J. (2013). Effects of exercise training on chronic inflammation in obesity : current evidence and potential mechanisms. *Sports Medicine*.

[21] Speretta G. F. F., Rosante M. C., Duarte F. O. (2012). The effects of exercise modalities on adiposity in obese rats. *Clinics*.

[22] Gielen S., Schuler G., Adams V. (2010). Cardiovascular effects of exercise training: molecular mechanisms. *Circulation*.

[23] Afzalpour M. E., Chadorneshin H. T., Foadoddini M., Eivari H. A. (2015). Comparing interval and continuous exercise training regimens on neurotrophic factors in rat brain. *Physiology & Behavior*.

[24] Qin F., Dong Y., Wang S. (2020). Maximum oxygen consumption and quantification of exercise intensity in untrained male Wistar rats. *Scientific Reports*.

[25] Graaf C. d., Donnelly D., Wootten D. (2016). Glucagon-likepeptide-1 and its class B G protein-coupled receptors: a long march to therapeutic successes. *Pharmacological Reviews*.

[26] Bhat G. A., Khan H. A., Alhomida A. S., Sharma P., Singh R., Paray B. A. (2018). GLP-I secretion in healthy and diabetic Wistar rats in response to aqueous extract of Momordica charantia. *BMC Complementary and Alternative Medicine*.

[27] Constans A., Pin-Barre C., Molinari F. (2021). High-intensity interval training is superior to moderate intensity training on aerobic capacity in rats: impact on hippocampal plasticity markers. *Behavioural Brain Research*.

[28] Puddu A., Sanguineti R., Montecucco F., Viviani G. L. (2014). Glucagon-likepeptide-1 secreting cell function as well as production of inflammatory reactive oxygen species is differently regulated by glycated serum and high levels of glucose. *Mediators of Inflammation*.

[29] Kamimura D., Ishihara K., Hirano T. (2003). IL-6 signal transduction and its physiological roles: the signal orchestration model. *Reviews of Physiology, Biochemistry & Pharmacology*.

[30] Makki K., Froguel P., Wolowczuk I. (2013). Adipose tissue in obesity-related inflammation and insulin resistance: cells, cytokines, and chemokines. *ISRN Inflamm*.

[31] Akbari M., Hassan-Zadeh V. (2018). IL-6 signalling pathways and the development of type 2 diabetes. *Inflammopharmacology*.

[32] Nauck M. A., Vardarli I., Deacon C. F., Holst J. J., Meier J. J. (2011). Secretion of glucagon-likepeptide-1 (GLP-1) in type 2 diabetes: what is up, what is down?. *Diabetologia*.

[33] Matos V. A. F., Souza D., Santos V. (2018). Acute effects of high-intensity interval and moderate-intensity continuous exercise on GLP-1, appetite and energy intake in obese men: a crossover trial. *Nutrients*.

[34] Hazell T. J., Townsend L. K., Hallworth J. R., Doan J., Copeland J. L. (2017). Sex differences in the response of total PYY and GLP-1 to moderate-intensity continuous and sprint interval cycling exercise. *European Journal of Applied Physiology*.

[35] Afrasyabi S., Marandi S. M., Kargarfard M. (2019). The effects of high intensity interval training on appetite management in individuals with type 2 diabetes: influenced by participants weight. *Journal of Diabetes and Metabolic Disorders*.

[36] Adam T. C., Westerterp-Plantenga M. S. (2004). Activity-induced GLP-1 release in lean and obese subjects. *Physiology & Behavior*.

[37] Pedersen B. K., Febbraio M. A. (2008). Muscle as an endocrine organ: focus on muscle-derivedinterleukin-6. *Physiological Reviews*.

[38] Motoyoshi S., Yamamoto Y., Munesue S. (2014). cAMP ameliorates inflammation by modulation of macrophage receptor for advanced glycation end-products. *Biochemical Journal*.

[39] Reynaert N. L., Gopal P., Rutten E. P., Wouters E. F., Schalkwijk C. G. (2016). Advanced glycation end products and their receptor in age-related, non-communicable chronic inflammatory diseases; Overview of clinical evidence and potential contributions to disease. *The International Journal of Biochemistry & Cell Biology*.

[40] Allen T. L., Whitham M., Febbraio M. A. (2012). IL-6 muscles in on the gut and pancreas to enhance insulin secretion. *Cell Metabolism*.

[41] Ellingsgaard H., Hauselmann I., Schuler B. (2011). Interleukin-6 enhances insulin secretion by increasing glucagon-likepeptide-1 secretion from L cells and alpha cells. *Nature Medicine*.

[42] Lim D. M., Park K. Y., Hwang W. M., Kim J. Y., Kim B. J. (2017). Difference in protective effects of GIP and GLP-1 on endothelial cells according to cyclic adenosine monophosphate response. *Experimental and Therapeutic Medicine*.

[43] Farinha J. B., Ramis T. R., Vieira A. F. (2018). Glycemic, inflammatory and oxidative stress responses to different high-intensity training protocols in type 1 diabetes: a randomized clinical trial. *Journal of Diabetes and Its Complications*.

[44] Boor P., Celec P., Behuliak M. (2009). Regular moderate exercise reduces advanced glycation and ameliorates early diabetic nephropathy in obese Zucker rats. *Metabolism*.

[45] Goldin A., Beckman J. A., Schmidt A. M., Creager M. A. (2006). Advanced glycation end products: sparking the development of diabetic vascular injury. *Circulation*.

[46] Gleeson M., Bishop N. C., Stensel D. J., Lindley M. R., Mastana S. S., Nimmo M. A. (2011). The anti-inflammatory effects of exercise: mechanisms and implications for the prevention and treatment of disease. *Nature Reviews Immunology*.

[47] Pramaningtyas M. D., Utoro T., Purwono S., Agustiningsih D. (2016). Changes on aortic diameter and number of Endothelin-B receptorin aortic endothelium of diabetes mellitus rat model after exercise. *Bangladesh Journal of Medical Science*.

[48] Chengji W., Xianjin F. (2018). Treadmill exercise alleviates diabetic cardiomyopathy by suppressing plasminogen activator inhibitor expression and enhancing eNOS in streptozotocin-induced male diabetic rats. *Endocrine Connections*.

[49] Kong X., Qu X., Li B. (2017). Modulation of low shear stressinduced eNOS multisite phosphorylation and nitric oxide production via protein kinase and ERK1/2 signaling. *Molecular Medicine Reports*.

[50] Grijalva J., Hicks S., Zhao X. (2008). Exercise training enhanced myocardial endothelial nitric oxide synthase (eNOS) function in diabetic Goto-Kakizaki (GK) rats. *Cardiovascular Diabetology*.

[51] Mahboob M., Rahman M. F., Grover P. (2005). Serum lipid peroxidation and antioxidant enzyme levels in male and female diabetic patients. *Singapore Medical Journal*.

[52] Mitranun W., Deerochanawong C., Tanaka H., Suksom D. (2014). Continuous vs interval training on glycemic control and macro- and microvascular reactivity in type 2 diabetic patients. *Scandinavian Journal of Medicine & Science in Sports*.

[53] Liu L., Yu B., Chen J. (2012). Different effects of intermittent and continuous fluid shear stresses on osteogenic differentiation of human mesenchymal stem cells. *Biomechanics and Modeling in Mechanobiology*.

[54] Wisloff U., Stoylen A., Loennechen J. P. (2007). Superior cardiovascular effect of aerobic interval training versus moderate continuous training in heart failure patients: a randomized study. *Circulation*.

[55] Lee L. S., Tsai M. C., Brooks D., Oh P. I. (2019). Randomised controlled trial in women with coronary artery disease investigating the effects of aerobic interval training versus moderate intensity continuous exercise in cardiac rehabilitation: CAT versus MICE study. *BMJ Open Sport Exerc Med*.

[56] Bedard K., Krause K. H. (2007). The NOX family of ROS-generating NADPH oxidases: physiology and pathophysiology. *Physiological Reviews*.

[57] Sarvasti D., Lalenoh I., Oepangat E., Purwowiyoto B. S., Santoso A., Romdoni R (2020). Cardiovascular protection variables based on exercise intensity in stable coronary heart disease patients after coronary stenting: a comparative study. *Vascular Health and Risk Management*.

[58] Gomez-Cabrera M. C., Vina J., Ji L. L. (2016). Role of redox signaling and inflammation in skeletal muscle adaptations to training. *Antioxidants*.

[59] Kesavulu M. M., Rao B., Giri R., Vijaya J., Subramanyam G., Apparao C. (2001). Lipid peroxidation and antioxidant enzyme status in Type 2 diabetics with coronary heart disease. *Diabetes Research and Clinical Practice*.

[60] Delbin M. A., Davel A. P. C., Couto G. K. (2012). Interaction between advanced glycation end products formation and vascular responses in femoral and coronary arteries from exercised diabetic rats. *PLoS One*.

[61] Patel S., Santani D. (2009). Role of NF-kappa B in the pathogenesis of diabetes and its associated complications. *Pharmacological Reports*.

[62] Ungvari Z., Orosz Z., Labinskyy N. (2007). Increased mitochondrial H_2_O_2_ production promotes endothelial NF-*κ*B activation in aged rat arteries. *American Journal of Physiology - Heart and Circulatory Physiology*.

[63] Eizadi M., Laleh B., Khorshidi D. (2018). The effect of aerobic training with difference durations on serum IL-10 in middle-aged obese females. *Acta Endocrinologica*.

[64] Kahkha H. M., Moazami M., Rezaeian N. (2020). The comparison of effect of high intensity interval training compared to aerobic training on serum levels of some of stressactivated protein kinases and glucose in type II diabetic men with peripheral neuropathy. *Journal of Critical Reviews*.

[65] Parameswaran N., Patial S. (2010). Tumor necrosis factor-alpha signaling in macrophages. *Critical Reviews in Eukaryotic Gene Expression*.

[66] Karstoft K., Pedersen B. K. (2016). Exercise and type 2 diabetes: focus on metabolism and inflammation. *Immunology & Cell Biology*.

[67] Cabral-Santos C., Gerosa-Neto J., Inoue D. S. (2015). Similar anti-inflammatory acute responses from moderate-intensity continuous and high-intensity intermittent exercise. *Journal of Sports Science and Medicine*.

[68] Lira F. S., dos Santos T., Caldeira R. S. (2017). Short-term high- and moderate-intensity training modifies inflammatory and metabolic factors in response to acute exercise. *Frontiers in Physiology*.

